# DRAM1 Regulates Autophagy Flux through Lysosomes

**DOI:** 10.1371/journal.pone.0063245

**Published:** 2013-05-17

**Authors:** Xing-Ding Zhang, Lin Qi, Jun-Chao Wu, Zheng-Hong Qin

**Affiliations:** Department of Pharmacology and Laboratory of Aging and Nervous Diseases, Soochow University School of Pharmaceutical Science, Suzhou, China; Wayne State University, United States of America

## Abstract

We have previously reported that the mitochondria inhibitor 3-nitropropionic acid (3-NP), induces the expression of DNA damage-regulated autophagy modulator1 (DRAM1) and activation of autophagy in rat striatum. Although the role of DRAM1 in autophagy has been previously characterized, the detailed mechanism by which DRAM1 regulates autophagy activity has not been fully understood. The present study investigated the role of DRAM1 in regulating autophagy flux. In A549 cells expressing wilt-type TP53, 3-NP increased the protein levels of DRAM1 and LC3-II, whereas decreased the levels of SQSTM1 (sequestosome 1). The increase in LC3-II and decrease in SQSTM1 were blocked by the autophagy inhibitor 3-methyl-adenine. Lack of TP53 or knock-down of TP53 in cells impaired the induction of DRAM1. Knock-down of DRAM1 with siRNA significantly reduced 3-NP-induced upregulation of LC3-II and downregulation of SQSTM1, indicating DRAM1 contributes to autophagy activation. Knock-down of DRAM1 robustly decreased rate of disappearance of induced autophagosomes, increased RFP-LC3 fluorescence dots and decreased the decline of LC3-II after withdraw of rapamycin, indicating DRAM1 promotes autophagy flux. DRAM1 siRNA inhibited lysosomal V-ATPase and acidification of lysosomes. As a result, DRAM1 siRNA reduced activation of lysosomal cathepsin D. Similar to DRAM1 siRNA, lysosomal inhibitors E64d and chloroquine also inhibited clearance of autophagosomes and activation of lysosomal cathapsin D after 3-NP treatment. These data suggest that DRAM1 plays important roles in autophagy activation induced by mitochondria dysfunction. DRAM1 affects autophagy through argument of lysosomal acidification, fusion of lysosomes with autophagosomes and clearance of autophagosomes.

## Introduction

3-nitropropionic acid (3-NP), a suicide inhibitor of the mitochondrial respiratory enzyme succinate dehydrogenase (SDH) [1], induces striatal cell death in vivo and in vitro [2]–[4]. When intoxicated in vivo, 3-NP produces symptoms and striatal neuronal loss in human brains replicating neuropathology of Huntington’s disease [4], [5]. We previously reported that intrastriatal administration of 3-NP induced TP53-dependent autpophagy activation and apoptosis. The TP53 specific inhibitor pifithrin-α (PFT-α) blocked induction of autophagic proteins including DNA Damage Regulated Autophagy Modulator1 (DRAM1), LC3-II and beclin1 and apoptotic proteins including TP53-upregulated modulator of apoptosis (PUMA) and BAX. Both pharmacological inhibitors of autophagy and caspases effectively inhibited 3-NP-induced cell death [6], [7].

DRAM1, a novel TP53 target gene, is an evolutionarily conserved lysosomal protein and has been reported to play an essential role in TP53-dependent autophagy activation and apoptosis [8]. The mechanism by which DRAM1 promotes autophagy is not clear. It is proposed that DRAM1 may exert its effects on autophagy through lysosomes, given the fact as a lysosomal membrane protein. Uncovering the molecular mechanism by which DRAM1 regulates autophagy would provide a better understanding of the role of TP53 signaling pathway in the regulation of cell death and survival.

Autophagy is a pathway delivering cytoplasmic components to lysosomes for degradation [9]–[13]. Macroautophagy involves the sequestration of a region of the cytoplasm in a double-membrane structure to form a unique vesicle called the autophagosome. Acidification of lysosomes is crucial for activation of cathepsins, fusion of lysosomes and autophagosomes and effective degradation of autophagic substrates. However, these late digestive steps of autophagy remain largely uncharacterized.

Lysosomes are cytoplasmic organelles that contain several enzymes mostly belonging to the hydrolases [14]. Internal pH of lysosomal is characteristically acidic and it is maintained around pH 4.5 by a proton pump, that transport H^+^ ions into lysosomes [15], [16]. Many autophagy inhibitors including the vinca alkaloids (e.g., vinblastine) and microtubule poisons that inhibit fusion of autophagosomes with lysosomes, inhibitors of lysosomal enzymes (e.g., leupeptin, pepstatin A and E64d), and compounds that elevate lysosomal pH (e.g., inhibitors of vacuolar-type ATPases, such as bafilomycin A1 and weak base amines including ammonia, methyl- or propylamine, chloroquine, and Neutral Red, some of which slow down fusion), act at the fusion and lysosomal degradation steps [17]. Lysosomal enzymes also play a role in activation of certain types of caspases and therefore, are involved in apoptosis [18]. Inhibition of lysosomes or lysosomal enzymes protects neurons against excitotoxicity and ischemic insults [19], [20]. Thus, it is of particularly interest to investigate if DRAM1 modulates autophagy through influencing lysosomal functions.

In this study, we report that 3-NP induced DRAM1-dependent stimulation of autophagy in A549 cell lines. DRAM1 promotes autophagy flux by enhancing lysosomal acidification.

## Materials and Methods

### Cell Lines and Reagents

A549 (TP53^+/+^) and H1299 (TP53^−/−^) and Hela cell lines were purchased from Shanghai Institute of Biochemistry and Cell Biology in China, and were grown at 37°C in 5% CO_2_ in RPMI supplemented with 2 mmol/L L-glutamine and 10% FCS. Primary mouse embryonic fibroblasts (MEFs) were derived from p53 wt and p53 KO sibling embryos, and maintained with DMEM supplemented with 10% FCS and antibiotics. 3-NP (N5636), 3-MA (M9281), carbonyl cyanide m-chlorophenylhydrazone (CCCP, C2759), ATP (A6559), chloroquine (C6628), E-64d (E8640) and Z-Vad-FMK (V116) were all purchased from Sigma-Aldrich (Saint Louis, MO, USA). LysoTracker Red (L7528) and LysoSentor (L7533) were purchased from Invitrogen-Molecular Probes (Shanghai, China). All cell culture reagents were purchased from Gibco (Gaithersburg, MD, USA) unless otherwise noted.

### Expression of GFP-LC3 and DRAM1-pEGFP

The activation of autophagy was detected following transfection of cells with GFP-LC3 and mRFP-GFP-LC3 expression plasmids (kindly provided by Dr. T. Yoshimori, National Institute of Genetics, Japan). The presence of several intense fluorescent dots in cells is indicative of the accumulation of autophagosomes. Transfection of cells with expression plasmids was performed using Lipofectamine 2000 (Invitrogen, 11668-019, Shanghai, China). For each condition, the number of GFP-LC3 dots per cell was determined with a fluorescence microscopy for at least 100 GFP-LC3-positive cells.

PcDNA4-DRAM1-His was generated by PCR from the I.M.A.G.E. clone for DRAM1 (Clone ID: NM_018370) with: CCCAAGCTTATGCTGTGCTTCCTGAGGGGAATG (forward) and CCGCTCGAGTCAAATATCACCATTGATTTCTGTG (reverse), and subsequently digested with BamH I and Xho I and cloned in to the BamH I and Xho I sites of pcDNA4/HisA (Invitrogen Carlsbad, CA, USA). pEGFP-N1-DRAM1 was generated through PCR primer: ATAGAATTCATGCTGTGCTTCCTGAGGGGA (forward) and CCGGGATCCTAATATCACCATTGATTTCTGTG(reverse), and products were T-A cloned in pMDTM19-T Vectors (Takara, D102A, Dalian, China) and digested with EcoR I and BamH I and cloned into pEGFP-N1 (Clonetech, D102A, Mountain View, CA, USA). Transfection of cells with expression plasmids was performed using Lipofectamine 2000 (Invitrogen, 11668-019, Shanghai, China).

### Knock-down of TP53 and DRAM1

Small interfering RNAs (siRNA) targeting the following mRNA: TP53, AAGACUCCAGUGGUAAUCUAC; DRAM1, (1) CCACGATGTATACAAGATA and (2) CCACAGAAATCAATGGTGA. Negative siRNA TAAGGCTATGAAGAGATAC, were synthesized by GenePharma (Shanghai, China). The siRNA oligos used to target DRAM1 genes were previously validated and described in the following articles [8], [21], [22]. For transfection, cells were plated in 9-cm dishes at 30% confluence, and siRNA duplexes (200 nM) were introduced into the cells using Lipofectamine 2000 (Invitrogen, 11668-019, Shanghai, China) according to the manufacturer’s recommendations.

### LC3 Immunofluorescence Assay

For immunofluorescence microscopic examination, cells were plated on 12-mm Poly-L-Lysine-coated cover slips and cultured for 24 h, then cells were treated with siRNA and drugs. Cells were washed in PBS, fixed with 4% paraformaldehyde in PBS at 4°C for 10 min, and then washed again with PBS. The cells were permeabilized with 0.25% Triton X-100, and were then blocked with 10% normal goat serum (NGS) for 15 min. Primary antibodies: a rabbit polyclonal antibody against LC-3 (Abgent, AJ1456c, Suzhou, China), a goat polyclonal antibody against cathepsin D (Santa Cruz, sc-6488, Santa Cruz, CA, USA) and a rabbit polyclonal antibody against LAMP2 (Abcam, ab37024, Cambridge, MA, USA) diluted in PBS were added to the cells and left for overnight at 4°C. The cover slips were washed three times before incubation with secondary antibodies using the same procedure as for the primary antibodies. The cover slips were mounted on slides with mounting medium (Sigma-Aldrich, F4680, Saint Louis, MO, USA) and were examined with a laser scanning confocal microscopy (Nikon, C1S1, Tokyo, Japan).

The pattern of distribution of exogenously expressed GFP-LC3 in A549 cells was observed with fluorescent microscopy. GFP-LC3 dot formation was quantified by counting 500 GFP-LC3-positive cells and expressed as the ratio of the number of cells with at least 5 GFP-LC3 dots and the number of GFP-LC3-positive cells. The assays were independently performed by two investigators in a blinded manner and similar results were obtained.

### Western Blot Analysis

Western blot analysis was performed as scribed previously [23]. Cells were harvested and rinsed twice with ice-cooled PBS and homogenized in a buffer containing 10 mmol/L Tris-HCl (pH 7.4), 150 mmol/L NaCl, 1% Triton X-100, 1% sodium deoxycholate, 0.1% SDS, 5 mol/L edetic acid, 1 mmol/L PMSF, 0.28 U/L aprotinin, 50 mg/L leupeptin, 1 mmol/L benzamidine, 7 mg/L pepstain A. Protein concentration was determined using the BCA kit. Thirty micrograms of protein from each sample was subjected to electrophoresis on 10–12% SDS-PAGE gel using a constant current. Proteins were transferred to nitrocellulose membranes and incubated with the Tris-buffered saline containing 0.2% Tween-20 (TBST) and 3% non-fat dry milk for 3 h in the presence of one of the following antibodies: a rabbit polyclonal antibody against LC-3 (Abgent, AJ1456c, San Diego, CA, USA), a mouse monoclonal antibody against TP53 (Cell Signaling Technology, 2524S, Boston, MA, USA), a mouse monoclonal antibody against β-actin (Santa Cruz, sc-58669), a goat polyclonal antibody against cathepsin D (Santa Cruz, sc-6488), rabbit polyclonal antibodies against DRAM1 (Stressgen, 905-738-100, Farmingdale, NY, USA), a rabbit polyclonal antibodies against SQSTM1 (Enzo Life Sciences, PW9860, Farmingdale, NY, USA),Membranes were washed and incubated with horseradish peroxidase-conjugated secondary antibodies in TBST containing 3% non-fat dry milk for 1 h. Immunoreactivity was detected with enhanced chemoluminescent autoradiography (ECL kit, Amersham, RPN2232, Piscataway, NJ, USA) according to the manufacturer’s instructions. The levels of protein expression were quantitatively analyzed with SigmaScan Pro 5. The results were normalized to loading control β-actin (Santa Cruz, sc-58669). DRAM1 peptide (Acris Antibodies, AP30304CP-N, San Diego, CA, USA) was used for evaluating the specificity of DRAM1 antibody. Pre-incubation of DRAM1 antibody with control peptide (1 µg control peptide/1 µL DRAM1 antibody) abolished binding activity of DRAM1 antibody (Figure S2).

### Determination of Lysosomal pH

For lysosomal pH estimation, A549 and Hela cells were seeded on circular glass cover slips and grown to conﬂuence in Dulbecco’s modified Eagle’s medium (DMEM) with 10% fetal bovine serum (FBS; Wisent, 080–150) at 37°C, 5% CO^2^. Lysosomes were loaded overnight with 70000 MW FITC-dextran (Sigma-Aldrich, 53471). and 0.5 mg/mL dextran-coupled Oregon Green 488 (Invitrogen-Molecular Probes, D-7173, Grand Island, NY, USA) in DMEM supplemented with 10% FBS, chased for 2 h at 37°C with 5% CO2 in DMEM (10% FBS) to allow complete transfer of dextrans to lysosomes, and washed to remove residual dextran. Non-attached cells were removed by rinsing with PBS and the cover slips were immediately placed in a cuvette filled with growth medium or PBS and pH was estimated from excitation ratio measurements as described previously [24]. The fluorescence emitted was recorded at two excitation wavelengths (440/490 nm for Oregon Green 488) using the largest excitation and emission slits by a scanning multiwell spectrophotometer (Ultra Micro- plate Reader; BIO-TEK Instruments, ELx800, Winooski, VT, USA). The pH values were derived from the linear standard curve generated via each fluorescent dextran in phosphate/citrate buffers of different pH between 3.5 and 7.5. The experiment was repeated six times.

### Spectrophotometric Measurement of H^+^ Transport

FITC-dextran loaded A549 and Hela cells were prepared as described above. After washing in PBS, cells were resuspended (10^8^ cells in 2 ml) in homogenization buffer (0.25 M sucrose, 2 mM EDTA, and 10 mM Hepes [pH 7.4]) and homogenized in a tight-fitting glass Dounce homogenizer. The homogenate was centrifuged (800 g, 10 min) to remove unbroken cells and the nuclei. The supernatant was centrifuged (6800 g, 10 min) to remove the large organelle such as mitochondrial. The supernatant was centrifuged (25000 g, 10 min) to obtain the light organelle including lysosomes. The precipitation layered over 10 ml of a 27% Percoll (Pharmacia Inc, 17-0891-01, New York, NY, USA) solution in homogenization buffer, underlayered with 0.5 ml of a 2.5 M sucrose solution. Centrifugation was done in a SW41Ti rotor (Beckman Instruments Inc, Brea, CA, USA) for 1.5 h at 35000 g. The layer of crude lysosomes of about 1.5 ml was collected at the bottom and then was centrifuged (100000 g, 60 min) to remove the other light organelle including mitochondrial at the bottom of the tube. Lysosomal fractions were equilibrated for up to 1 h in 125 mM KCl, 1 mM EDTA, and 20 mM Hepes (pH 7.5). Fluorescence was recorded continuously with excitation at 490 nm and emission at 520 nm. Upon addition of ATP (Sigma-Aldrich, A6559, Saint Louis, MO, USA), a progressive decrease in fluorescence intensity was observed, indicative of intralysosomal acidification [25]. As expected, the pH gradients in both samples were collapsed by the addition of the bafilomycin A1 (1 µM) (Sigma-Aldrich, B1793). The solvents alone had no effect on lysosomal pH. The reagents used and their final concentrations were: ATP (K^+^ salt, pH 7.5, 5 mM), bafilomycin A1 (1 µM).

### Statistical Analysis

Statistical analysis was carried out by one-way analysis of variance (ANOVA) followed by Dunnett t-test or multiple means comparisons by Tukey’s test. Differences were considered significant when p<0.05.

## Results

### 3-NP Induces Autophagy Activation

**Figure 1:**
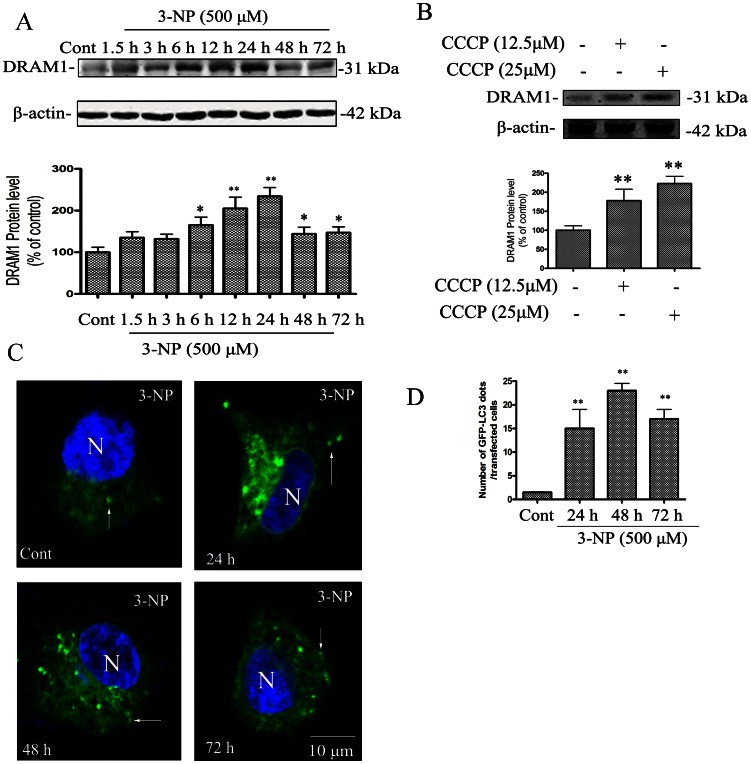
3-NP activated autophagy. A549 cells were treated with 3-NP (500 µM) and harvested 24, 48 and 72 h later. (A) Immunoblot analysis of DRAM1 levels in A549 cells under conditions of: no treatment (Ctrl) and 3, 6, 12, 24, 48 and 72 h after 3-NP. (B) Immunoblot analysis of DRAM1 levels in A549 cells under conditions of: no treatment (Ctrl) and 12.5µM and 25 µM of CCCP treatment for 4 h. Bars represent mean±SE; n = 4. Statistical comparisons were carried out by ANOVA followed by Dunnett t-test. **P<0.01 (3-NP group vs. control group). (C) Representative images of GFP-LC3 fluorescence in cells transfected with GFP-LC3 plasmid 24, 48 and 72 h after 3-NP (500 µM). N: the nucleus. Thin arrows: GFP-LC3 dots. The scale bar represents 10 µm. (D) Quantitative analysis of the number of GFP-LC3 puncta. Number of cells with GFP-LC3 dots was scored in 100 GFP-LC3-positive cells. Statistical comparisons were carried out by ANOVA followed by Dunnett t-test. **P<0.01 (3-NP group vs. control group).

**Figure 2:**
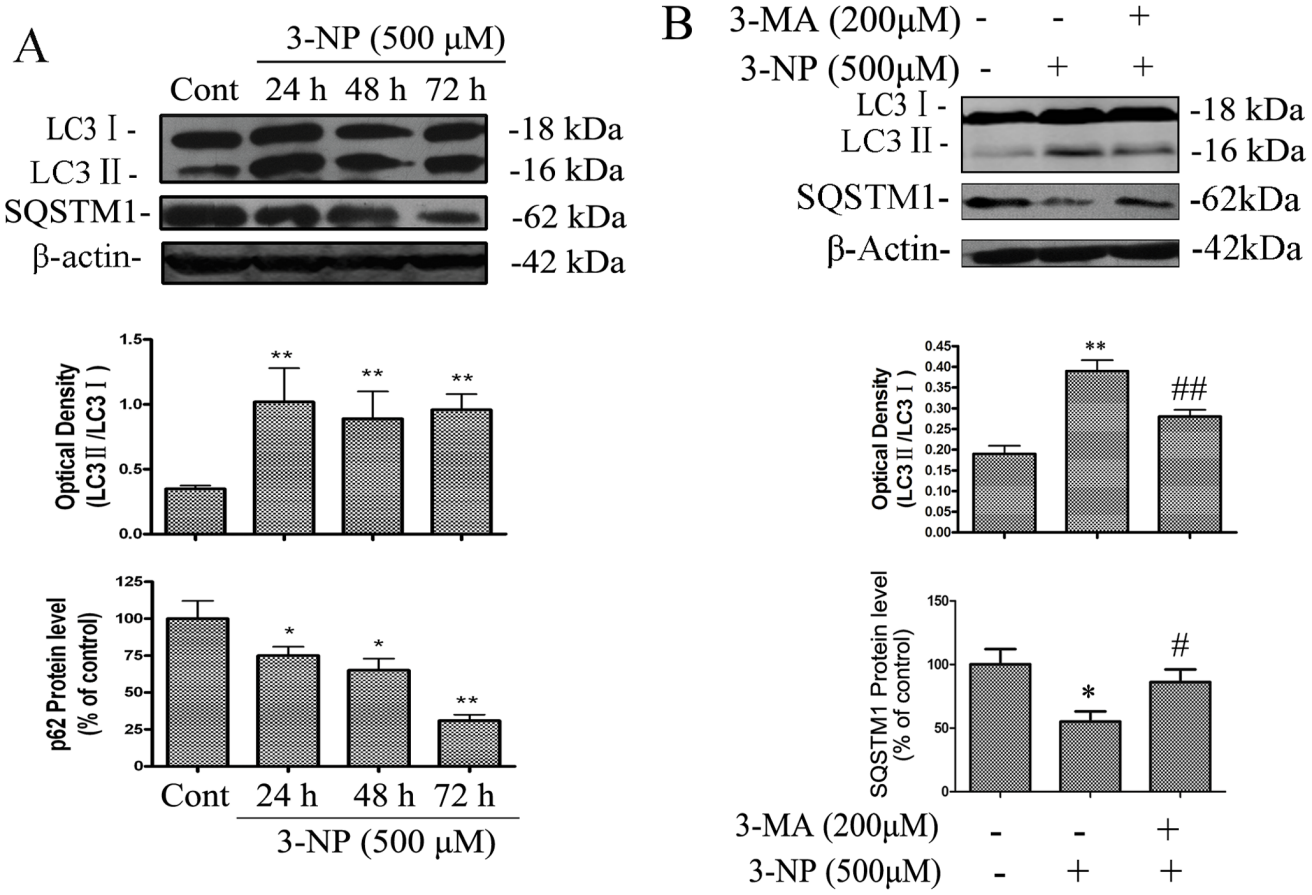
Autophagy was induced by 3-NP and blocked by 3-MA. (A) Immunoblot analysis of LC3 and SQSTM1 levels in A549 cells under conditions of: no treatment (Ctrl) and 24, 48 and 72 h after 3-NP. Protein extracts were subjected to SDS-PAGE and immunoblotting. Densities of protein bands were analyzed with an image analyzer (SigmaScan Pro 5) and normalized to the loading control (β-actin). The data are expressed as percentage of control (untreated cells). Bars represent mean±SE; n = 4. (B) Immunoblot analysis of LC3 and SQSTM1 levels in cells under conditions of: no treatment (Cont), 3-NP (500 µM) and 3-MA (200 µM) +3-NP (500 µM). Protein extracts were subjected to SDS-PAGE and immunoblotting. Densities of protein bands were analyzed with an image analyzer (SigmaScan Pro 5) and normalized to the loading control (β-actin). The data are expressed as percentage of control (untreated cells). Bars represent mean±SE; n = 4. Statistical comparisons were carried out by ANOVA followed by Dunnett t-test. *P<0.05 (3-NP group vs. control group). #P<0.05 (3-MA +3-NP- treated group vs. 3-NP- treated group). **P<0.01 (3-NP group vs. control group). ##P<0.05 (3-MA +3-NP- treated group vs. 3-NP- treated group).

The present study examined if autophagic and apoptotic pathways are activated in A549 cells after 3-NP treatment. The results showed that 3-NP-induced a significant increase in the protein levels of DRAM1 from 3 to 72 h, with a peak induction at 24 h after 3-NP treatment (Figure 1A). The specificity of DRAM1 antibody was checked with Western blot analysis and immunofluorescence assay using DRAM1 control peptide (Figure S2). To further test if mitochondria respiration failure triggers DRAM1 expression, we used CCCP to uncouple mitochondria oxidation and phosphorylation, the results showed that CCCP significantly increased the DRAM1 protein levels (Figure 1B). LC3 is a mammalian homologue of yeast Atg8p and LC3-II is required for the formation of autophagosomes [26]. As shown in Fig. 1C, 3-NP induced a time-dependent increase in GFP-LC3 in A549 cells, and LC3-positive vesicular profiles of sizes 0.5–2.0 µm were significantly more numerous in 3-NP-treated cells 48 h after treatment (Figure 1C and 1D). To provide biochemical evidence of autophagy activation, the time-course of 3-NP-induced changes in LC3-II in A549 cells was determined 24 to 72 h after 3-NP (500 µM) treatment. The expression of LC3-II significantly increased 24 h after 3-NP treatment (Figure 2A). As an additional assessment of autophagy activity, the degradation of SQSTM1 (sequestosome 1), an autophagy substrate, was determined [27]. The present results showed that the protein level of SQSTM1 decreased 24–72 h after 3-NP treatment (Figure 2A). As a confirmation of autophagy activation, the present study demonstrated that the elevation of LC3- II and the decline of SQSTM1 were blocked by the autophagy inhibitor 3-methyl-adenine (Figure 2B).

**Figure 3:**
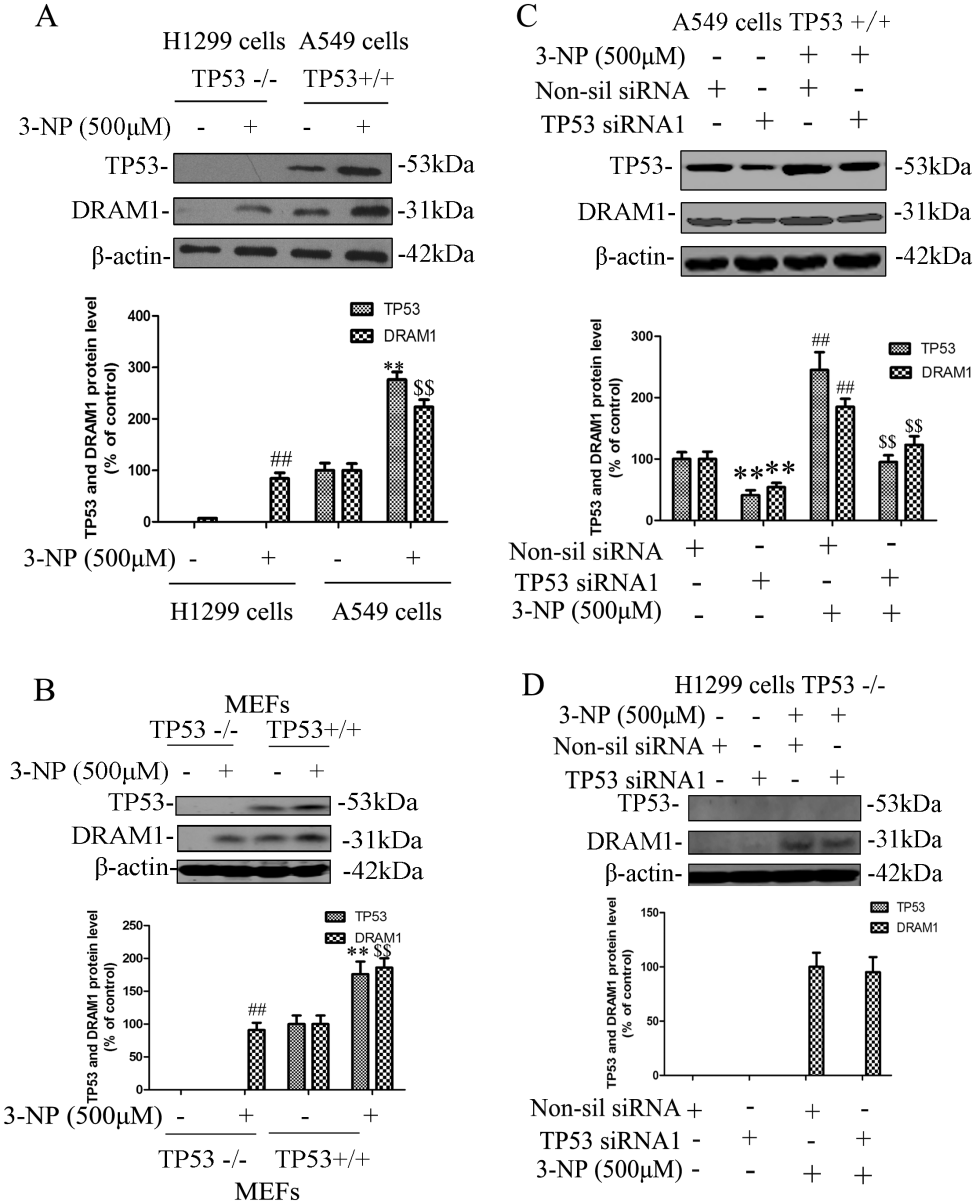
TP53 dependency of DRAM1 induction after 3-NP treatment. A549 and H1299 cells were treated with 3-NP (500 µM) and harvested 48 h later. (A) Immunoblot analysis of TP53 and DRAM1 levels in A549 and H1299 cells under conditions of: no treatment (Ctrl) and 48 h after 3-NP. Protein extracts were subjected to SDS-PAGE and immunoblotting. Densities of protein bands were analyzed with an image analyzer (SigmaScan Pro 5) and normalized to the loading control (β-actin). The data are expressed as percentage of control (untreated cells). Bars represent mean±SE; n = 4. Statistical comparisons were carried out by ANOVA followed by Dunnett t-test. **P<0.01 (3-NP group vs. control group). ^##^P<0.01 (3-NP group vs. control group). ^$$^P<0.01 (3-NP group vs. control group). (B) Immunoblot analysis of TP53 and DRAM1 levels in p53 wt and p53 KO MEFs under conditions of: no treatment (Ctrl) and 48 h after 3-NP. Protein extracts were subjected to SDS-PAGE and immunoblotting. Densities of protein bands were analyzed with an image analyzer (SigmaScan Pro 5) and normalized to the loading control (β-actin). The data are expressed as percentage of control (untreated cells). Bars represent mean±SE; n = 4. Statistical comparisons were carried out by ANOVA followed by Dunnett t-test. **P<0.01 (3-NP group vs. control group). ^##^P<0.01 (3-NP group vs. control group). ^$$^P<0.01 (3-NP group vs. control group). (C) A549 cells were transfected with TP53 siRNA or a non-silencing siRNA. Forty-eight hours after transfection of cells with TP53 siRNA, cells were harvested and protein levels of TP53 and DRAM1 were analyzed with immunoblotting 24 h after 3-NP. Densities of protein bands were analyzed with Sigma Scan Pro 5 and normalized to the loading control (β-actin). The data are expressed as percentage of control. Bars represent mean±SE; n = 4. Statistical comparisons were carried out by ANOVA followed by Dunnett t-test. **P<0.01 TP53 siRNA group vs. non-silencing siRNA group. (D) H1299 cells were transfected with TP53 siRNA or a non-silencing siRNA. Forty-eight hours after transfection of cells with TP53 siRNA, cells were harvested and protein levels of TP53 and DRAM1 were analyzed with immunoblotting 24 h after 3-NP. Densities of protein bands were analyzed with Sigma Scan Pro 5 and normalized to the loading control (β-actin). The data are expressed as percentage of control. Bars represent mean±SE; n = 4. Statistical comparisons were carried out by ANOVA followed by Dunnett t-test.

It was reported that DRAM1 is a TP53 target gene. We determined the TP53 dependency in 3-NP-induced DRAM1 expression. In H1299 cells which lack of TP53, 3-NP only slightly induced DRAM1 expression, while in A549 cells which express wt TP53, 3-NP robustly induced the expression of DRAM1 (Figure 3A). The similar results were seen in TP53 wt and TP53 null MEFs cells (Figure 3B). Treatment of A549 cells with TP53 siRNA, partially inhibited both basal and 3-NP-induced the expression of DRAM1 (Figure 3C). In contrast, treatment of H1299 with TP53 siRNA did not block 3-NP-induced expression of DRAM1 (Figure 3D). These results suggest that induction of DRAM1 largely depends on TP53 mechanism, but other signaling pathways are also be involved in regulating DRAM1 expression after 3-NP treatment [28].

### DRAM1 Mediates Autophagy Activation

**Figure 4:**
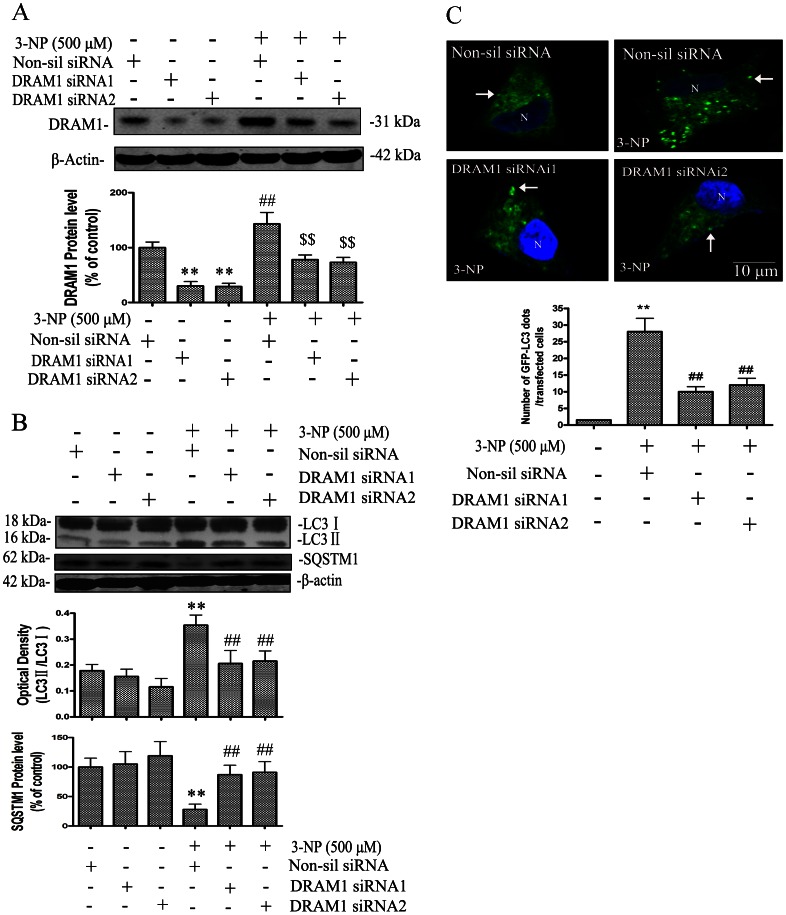
DRAM1 mediated autophagy activation. (A, B) A549 cells were transfected with DRAM1 siRNA or a non-silencing siRNA. Left: Forty-eight hours after transfection of cells with DRAM1 siRNA, cells were harvested and protein levels of DRAM1, LC3 and SQSTM1 were analyzed with immunoblotting. Right: Twenty-four hours after transfection of cells with DRAM1 siRNA, cells were treated with 3-NP (500 µM). Cells were harvested and protein levels of LC3 and SQSTM1 were analyzed with immunoblotting 24 h after 3-NP. Densities of protein bands were analyzed with SigmaScan Pro 5 and normalized to the loading control (β-actin). The data are expressed as percentage of control (non-silencing siRNA group). Bars represent mean±SE; n = 4. Statistical comparisons were carried out by ANOVA followed by Dunnett t-test. **P<0.01 non-silencing siRNA group vs. control group. ^##^P<0.01 DRAM1 siRNA group vs. non-silencing siRNA group. (C) Representative images of GFP-LC3 fluorescence in cells transfected with GFP-LC3 and treated with DRAM1 siRNAs in the presence or absence of 3-NP (500 µM). Number of cells with GFP-LC3 dots was scored in 100 GFP-LC3-positive cells. N: the nucleus. Thin arrows: GFP-LC3 dots. The scale bar represents 10 µm. Bars represent mean±SE; n = 4. Statistical comparisons were carried out by ANOVA followed by Dunnett t-test. **P<0.01 (siRNA group vs. non-silencing siRNA group).

To understand the role of DRAM1 in the regulation of autophagy, the present study investigated the role of DRAM1 in autophagy activation in response to 3-NP treatment in A549 and Hela cells. Knock-down of DRAM1 using siRNA significantly reduced the expression of DRAM1 proteins in A549 cells (Figure 4A) and in Hella cells (Figure S1 A). After knock-down of DRAM1 with siRNA, the basal expression and induction of LC3-II by 3-NP was markedly reduced in both A549 cells (Figure 4B) and Hela cells (Figure S1 **A**). In addition, 3-NP-induced reduction of SQSTM1 was blocked by DRAM1 siRNA in A549 cells (Figure 4B). The formation of GFP-LC3 puncta after 3-NP treatment was also inhibited in the presence of DRAM1 siRNA in A549 cells (Figure 4C) and in Hela cells (Figure S1 B). In addition to inhibiting the production of LC3-II, SQSTM1 levels increased in DRAM1 siRNA-treated cells (Figure 4B). These lines of evidence support an important role of DRAM1 in autophagy activation.

### DRAM1 Enhances Autophagosomes Clearance

**Figure 5:**
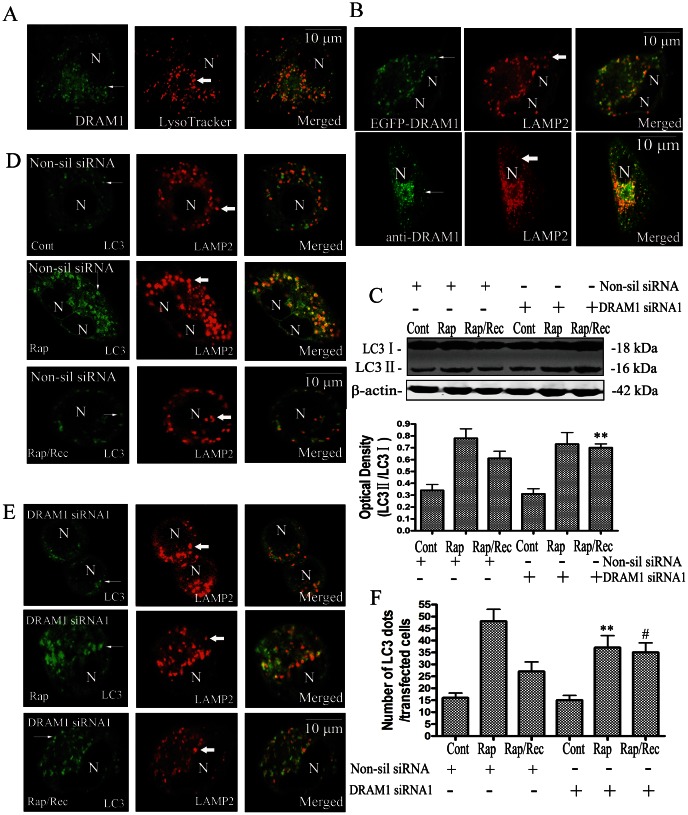
Knock-down of DRAM1 impaired the clearance of autophagosomes. (A) DRAM1 was predominantly localized in lysosomes (Lysotraker). A549 cells were transfected with GFP-DRAM1 for 48 h. Cells were incubated with LysoTracker (0.5 µM) and co-localization of DRAM1-GFP (green) and the LysoTracker (red) was assessed with a confocal microscopy. N: the nucleus. Thin arrows: GFP-DRAM1 fluorescence. Thick arrows: LysoTracker. (B) DRAM1 was predominantly localized in lysosomes (LAMP2). Up panel: A549 cells were transfected with GFP-DRAM1 for 48 h. Cells were processed for immunofluorescence using LAMP2 antibodies and co-localization of DRAM1-GFP (green) and the LAMP2 (red) was assessed with a confocal microscopy. N: the nucleus. Thin arrows: GFP-DRAM1 fluorescence. Thick arrows: LAMP2. Low panel: A549 cells were processed for immunofluorescence using DRAM1 and LAMP2 antibodies, and co-localization of DRAM1 (green) and the LAMP2 (red) was assessed with a confocal microscopy. N: the nucleus. Thin arrows: anti-DRAM1 fluorescence. Thick arrows: LAMP2. (C) Immunoblot analysis of LC3 levels in A549 cells under conditions: untreated (Cont), rapamycin (Rap) treatment, and 6 h after rapamycin removal (Rap/Rec). Densities of protein bands were analyzed with an image analyzer (SigmaScan Pro 5) and normalized to the loading control (β-actin). The data are expressed as percentage of control (non-silencing siRNA group). Bars represent mean±SE; n = 4. Statistical comparisons were carried out by ANOVA followed by Tukey’s test. **P<0.01 (DRAM1 siRNA treatment group vs. non-silencing siRNA group). (D) A549 cells were analyzed with double-immunofluorescence using LC3 and LAMP2 antibodies in the presence of rapamycin and 6 h after removal of rapamycin. N: the nucleus. Thin arrows: dots of LC3 immonureactivity. Thick arrows: LAMP2. The scale bar represents 10 µm. (E) DRAM1 siRNA-treated cells were analyzed with double-immunofluorescence using LC3 and LAMP2 antibodies in the presence of rapamycin and 6 h after removal of rapamycin. N: the nucleus. Thin arrows: dots of GFP-LC3 fluorescence. Thick arrows: LAMP2. The scale bar represents 10 µm. (F) In cells after DRAM1 siRNA treatment, the number of LC3 dots was scored in 100 GFP-LC3-positive cells in the presence or absence of 3-NP. The data are expressed as percentage of control. Bars represent mean±SE; n = 4. Statistical comparisons were carried out by Tukey’s test. **P<0.01 (DRAM1 siRNA treatment group vs. non-silencing siRNA group). ^#^P<0.05 (DRAM1 siRNA treatment group vs. non-silencing siRNA group).

To study the mechanisms of DRAM1 in regulating autophagy, A549 cells were transfected with GFP-DRAM1. The lysosomal localization of DRAM1 was examined with LysoTracker and LAMP2 immunofluorescence or double immunofluorescence of DRAM1 and LAMP2. LysoTracker is a commonly used lysosomal probe because it is an acidotropic fluorescent dye for labeling and tracking acidic organelles in live cells. Marked co-localization of DRAM1 and LysoTracker (Figure 5A) or DRAM1 and LAMP2 (Figure 5B) was seen with a confocal microscopy. The quantitative analysis revealed that colocalization of DRAM1 puncta and LAMP2 was 74.8±5.6% (data not shown), suggesting that DRAM1 predominantly localizes to lysosomes. The clearance of autophagosomes is a measure of autophagy flux. In control cells, acute autophagy induction with rapamycin elevated LC3-II levels as revealed by immunoblotting. After removing rapamycin from the medium for 6 h, LC3-II returned towards baseline levels. While in DRAM1 siRNA-treated cells, LC3-II remained elevated 6 h after removing rapamycin (Figure 5C). Double immunofluorescence of LC3 and LAMP2 demonstrated the formation of large number of LC3-LAMP2-positive vesicles in siRNA untreated cells after rapamycin exposure. Treatment of cells with DRAM1 siRNA reduced the number of LC3-LAMP2-posive vesicles (Figure 5D). After removal of rapamycin for 6 h, a number of LC3-LAMP2-positive vesicles were cleared in siRNA untreated cells but more LC3-LAMP2-positive vesicles remained in the cells treated with DRAM1 siRNA (Figure 5E and 5F). These suggest that both the formation and the clearance of autophagic vacuoles are impaired in DRAM1 siRNA-treated A549 cells.

### DRAM1 Affects Lysosomal Degradation and Lysosomal Acidification

**Figure 6:**
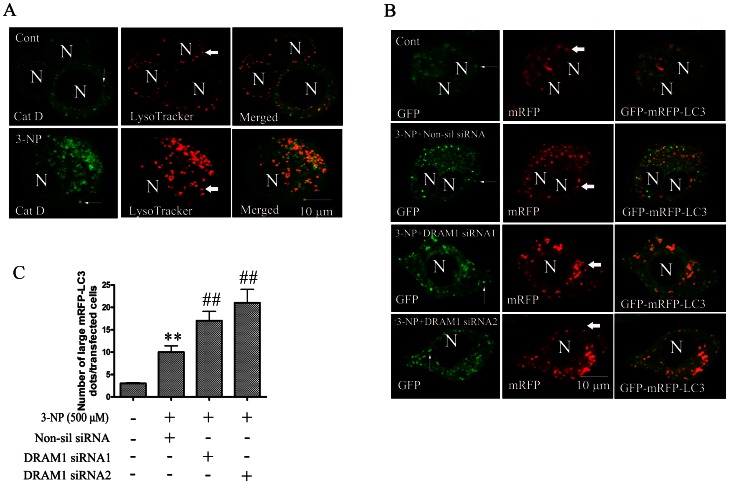
Knock-down DRAM1 inhibited autophagosome maturation process. (A) Lysosomes were activated by 3-NP. A549 cells were treated with 3-NP (500 µM) for 48 h. Cells were incubated with LysoTracker (0.5 µM) and processed for immunofluorescence using Cathepsin D (Cat D) antibodies. The co-localization of Cat D (green) and the LysoTracker (red) was assayed by confocal microscopy. N: the nucleus. Thin arrows: Cat D immunoreactivity. Thick arrows: LysoTracker. The scale bar represents 10 µm. (B) Accumulation of mRFP-LC3 in DRAM1 siRNA-treated cells. Representative images of mRFP-GFP-LC3 fluorescence in cells transfected with mRFP-GFP-LC3 and treated with DRAM1 siRNAs in the presence or absence of 3-NP (500 µM). N: the nucleus. Thin arrows: GFP-LC3 dots. Thick arrows: mRFP-LC3 dots. The scale bar represents 10 µm. (C) Number of cells with GFP-LC3 dots was scored in 100 GFP-LC3-positive cells. Statistical comparisons were carried out by ANOVA followed by Dunnett t-test. **P<0.01 non-silencing siRNA group vs. control group. ^##^P<0.01 DRAM1 siRNA group vs. non-silencing siRNA group.

Lysosomal enzyme, cathepsin D, plays an essential role in the degradation process of autophagic activity. The present study employed double immunofluorescence of cathepsin D and LysoTracker to explore the role of DRAM1 in lysosomal function. We observed that cathepsin D was virtually confined in LysoTracker fluorescence-positive vesicles in A549 cells. 3-NP treatment increased the expression of cathepsin D and the number of LysoTraker labeled lysosomes (Figure 6A).

GFP-LC3 is the most widely used marker for autophagosomes. When localized to autolysosomes, GFP-LC3 loses fluorescence due to lysosomal acidic and degradative conditions. While mRFP-LC3 is more stable in acidic conditions and fluorescence remains after fusion of autophagosomes with lysosomes. Thus, we used mRFP-GFP tandem fluorescent-tagged LC3 to monitor the process of autophagy maturation [29]. The result showed that 3-NP increased the expression of LC3, most of LC3 displayed yellow color due to emitted both GFP and RFP fluorescence. However, due to stronger fluorescence of GFP than that of RFP, some green LC3 patches were also observed. Knock-down of DRAM1 with siRNA slightly reduced GFP-LC3 fluorescence (reflecting attenuation of autophagy induction), but robustly increased the number of large mRFP-LC3 puncta (Figure 6B and 6C). In the condition of treatment with 3-NP in the presence of non-sil siRNA, yellow punctas were few because degradation of autolysosomes was smooth. While in the condition of treatment with 3-NP in the presence of DRAM1 siRNA, more large yellow pinctas were observed (Figure 6B). These results indicate that the clearance of autophagic vacuoles is impaired in DRAM1 siRNA-treated A549 cells.

**Figure 7:**
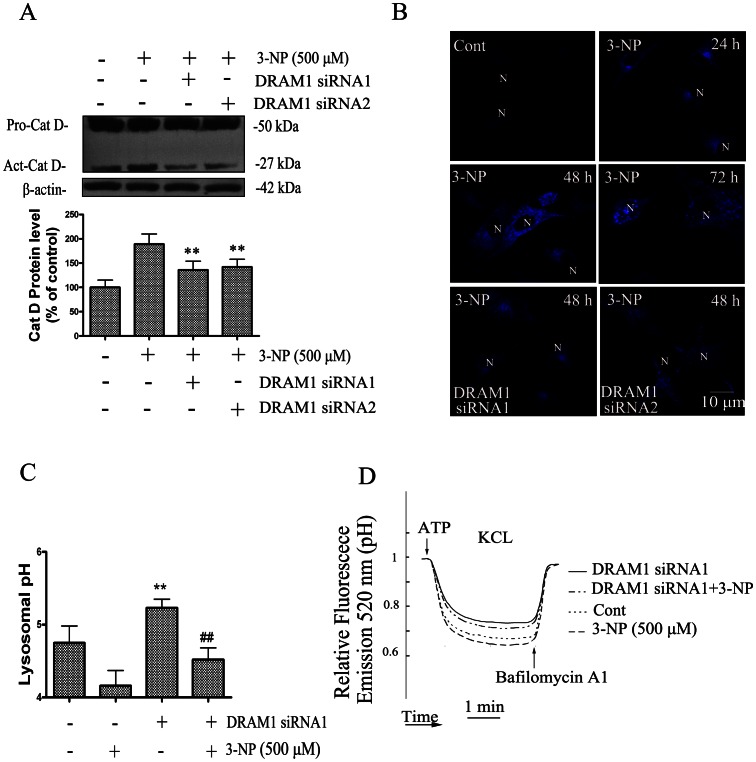
Knock down DRAM1 inhibited lysosomal acidification and cathepsin D activation. (A) A549 cells were transfected with DRAM1 siRNA or a non-silencing siRNA. Left: Forty-eight hours after transfection of DRAM1 siRNA, cells were harvested and protein levels of cat D were analyzed with immunoblotting. Right: Twenty-four hours after transfection of cells with DRAM1 siRNA, cells were treated with 3-NP (500 µM) for 24 h. Cells were harvested and protein levels of cat D were analyzed with immunoblotting. Densities of protein bands were analyzed with SigmaScan Pro 5 and normalized to the loading control (β-actin). The data are expressed as percentage of control (non-silencing siRNA cells). Bars represent mean±SE; n = 4. Statistical comparisons were carried out by ANOVA followed by Dunnett t-test. **P<0.01 (DRAM1 siRNA group vs. non-silencing siRNA group). (B) Lysosomal acidification was measured using LysoSensor DND-167. In control cells, the fluorescence of LysoSensor was measured from 24 to 72 h, and in DRAM siRNA-treated cells the fluorescence was measured in 48 h after transfection of DRAM1 siRNA. N: the nucleus. The scale bar represents 10 µm. (C) Lysosomal pH was measured ratio-metrically using fluorescent dextrans. WT cells and DRAM1 siRNA1-treated cells were loaded with the pH-sensitive fluorescent dextrans by endocytosis for 1 h at 37°C and then subjected to pulse-chase assay in the presence or absence of the 3-NP (500 µM). Lanes 2 and 4 depict pH values obtained with FITC-dextran after the addition of 500 nM 3-NP. The data are expressed as percentage of control (non-silencing siRNA cells). Bars represent mean±SE; n = 4. Statistical comparisons were carried out by ANOVA followed by Dunnett t-test. **P<0.01 (DRAM1 siRNA group vs. non-silencing siRNA group). ^##^P<0.01 (DRAM1 siRNA group vs. non-silencing siRNA group with 3-NP treatment). (D) Lysosomal V-ATPase activity was inhibited in DRAM1 siRNA1-treated cells. Lysosomes from control cells and DRAM1 siRNA1-treated cells were loaded with FITC-dextran (molecular weight 70,000). A549 cells were then homogenized and used for in vitro-acidification assays. Fluorescence was recorded continuously with excitation at 490 nm and emission at 520 nm. Upon addition of ATP, a progressive decrease in fluorescence intensity was observed, indicative of intralysosomal acidification. The decrement was reversed by bafilomycin A1, a V-ATPase inhibitor.

As most lysosomal cathepsins work at acidic pH, the effect of DRAM1 silencing on activation of cathepsin D was examined. The results of immunoblotting showed that knock-down of DRAM1 significantly inhibited 3-NP-induced production of the active form of cathepsin D (Figure 7A), suggesting activation of cathepsin D is compromised. To assess lysosomal acidification, we used LysoSensor DND-167. The LysoSensor dye is an acidotropic probe that appears to accumulate in acidic organelles as the result of protonation. In control cells, the fluorescence of LysoSensor was enhanced from 24 to 72 h after 3-NP exposure. By contrast, in DRAM1 siRNA-treated cells, the fluorescence was lower than that in the control cells (Figure 7B). We further measured lysosomal pH in quantization. The cells were loaded with the pH-sensitive reporter FITC-dextran by endocytosis for 1 h and then chased in the control and DRAM1 siRNA-treated cells in the presence and absence of 3-NP. WT cells exhibited an intralysosomal pH of 4.75, and lysosomal pH decreased following 3-NP treatment (Figure 7C). In contrast, the lysosomal pH values decreased to a lesser extent (5.23) in DRAM1 siRNA-treated cells following 3-NP treatment in both A549 cells (Figure 7C) and in Hela cells (Figure S1 C). These results suggest that there is a defective lysosomal acidification in DRAM1 siRNA-treated cells. Lysosomal acidification requires the activity of the ATP-dependent vacuolar proton pump [30]. We examined the ATP-dependent lysosomal acidification using the pH sensitive dye FITC-dextran. This dye accumulates inside lysosomes due to its weak basic net charge in response to ATP addition. As shown in Figure 7D, addition of ATP caused a dramatic drop in FITC fluorescence as a result of lysosomal acidification in control and 3-NP-treated cells. In DRAM1 siRNA-treated cells, ATP-induced drop in fluorescence emission was reduced, reflecting a reduction in internal lysosomal acidification. Reduction in FITC fluorescence by ATP was inhibited by the V-ATPase inhibitor bafilomycin A1. The similar results were obtained in Hela cells (Figure S1 D). Thus, the impairment of acidification in DRAM1 siRNA-treated cells might be due to a decrease in V-ATPase activity.

**Figure 8:**
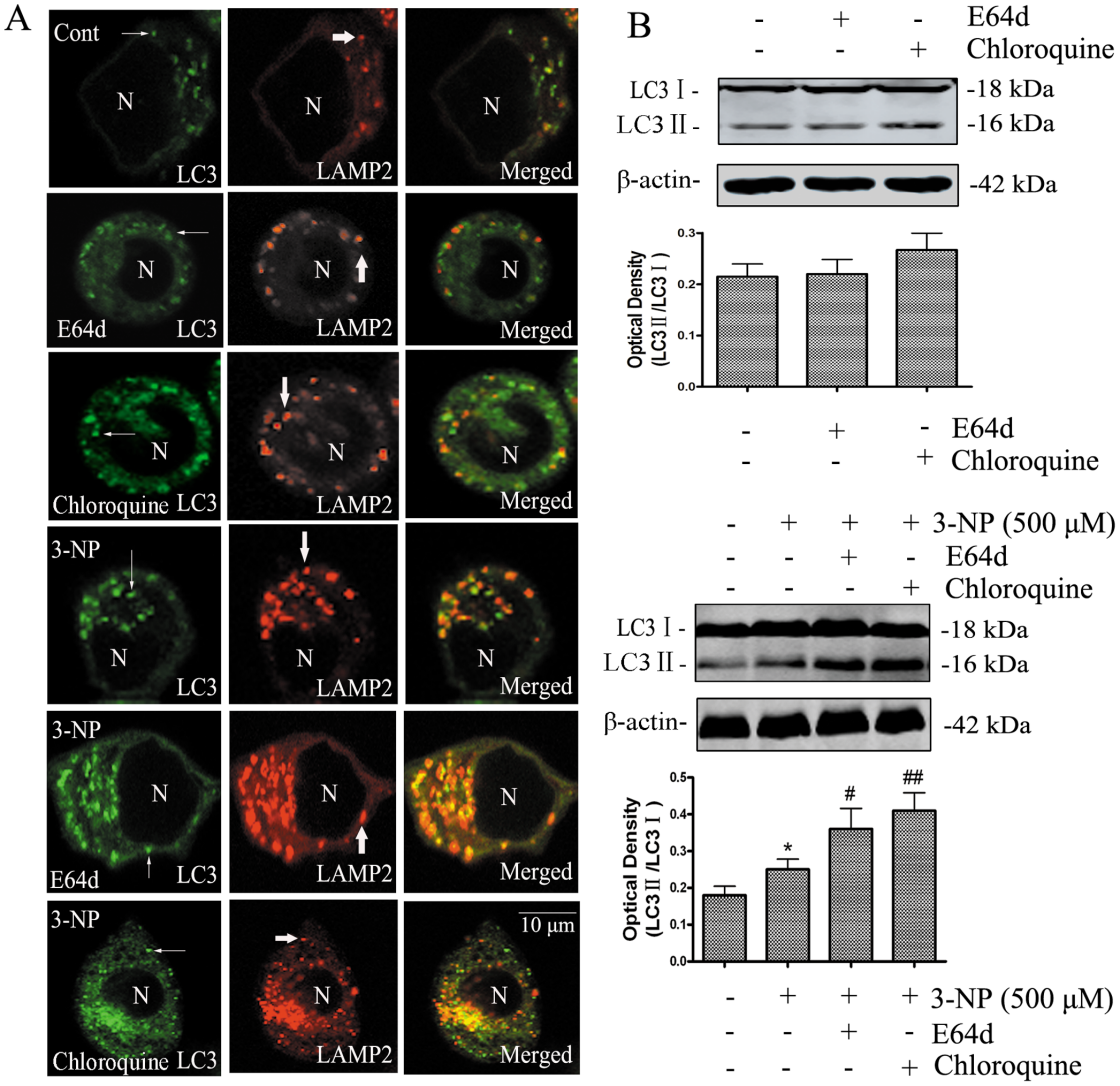
Lysosomal inhibitors inhibited autophagosome clearance. (A) Accumulation of autophagosomes was analyzed with double-immunofluorescence using antibodies against LC3 and LAMP2 after E64d (10 µM) or chloroquine (20 µM) treatment for 24 h in the presence or absence of 3-NP (500 µM). N: the nucleus. Thin arrows: dots of LC3 immunoreactivity. Thick arrows: LAMP2 immunoreactivity. The scale bar represents 10 µm. (B) Immunoblot analysis of LC3-II levels in cells under conditions of: no treatment (Cont), E64d (10 µM), chloroquine (20 µM), 3-NP (500 µM), E64d (10 µM) +3-NP (500 µM) or chloroquine (20 µM) +3-NP (500 µM). Cells were harvested for immunoblotting 48 h after 3-NP treatment. Densities of protein bands were analyzed with SigmaScan Pro 5 and normalized to the loading control (β-actin). The data are expressed as percentage of control (untreated cells). Bars represent mean±SE; n = 4. Statistical comparisons were carried out by ANOVA followed by Dunnett t-test. *P<0.05 (3-NP treated group vs. control group). ^#^P<0.05 (E64d+3-NP- or chloroquine +3-NP-treated group vs. 3-NP- treated group). ^##^P<0.01 (E64d plus 3-NP or chloroquine plus 3-NP treatment group vs. 3-NP treatment group).

Foregoing observations indicate that DRAM1 regulates autophagy flux mainly thought lysosomes. Thus, the lysosomal inhibitors E64d (10 µM) and chloroquine (20 µM) were used to evaluate if inhibition of lysosomal functions produces effects similar to knock-down of DRAM1. Many autophagy inhibitors act on post-sequestration steps and agents, such as bafilomycin A1, that blocks autophagy activity are known to cause accumulation of autophagosomes [31]. Chloroquine is a compound that elevates lysosomal pH, and E64d is an effective inhibitor of lysosomal enzymes [32]. After 3-NP treatment, more LAMP2-positive vacuoles were observed. Compared with cells treated with 3-NP alone, LC3 in E64d or chloroquine-treated cells accumulated more LAMP2-positive vacuoles (Figure 8A). As shown in Fig. 8B, LC3-II accumulated after E64d or chloroquine treatment. These results suggest a defective clearance of autophagic vacuoles in E64d- and chloroquine-treated cells.

## Discussion

3-NP acts as an irreversible inhibitor of succinate dehydrogenase and thus results in an impairement of energy metabolism, oxidative stress and activation of glutamate receptors [33]. Mitochondria are important intracellular organelles and the collapse of mitochondria membrane potential may be a signal for activation of autophagy and apoptosis. Previous in vivo studies suggest that 3-NP-induced cell death in rat striatum involves TP53-dependent activation of apoptosis and autophagy [6]. It was also reported that DRAM1 and SQSTM1 regulated cell migration and invasion of glioblastoma stem cells [34]. TP53 target gene DRAM1 possibly mediates down stream multiple functions in autophagy and cell death. The present in vitro studies found that 3-NP inhibited cell viability of A549 cells at the doses of 250 µM to 1 mM (data not shown). The activation of autophagy was demonstrated by increases in LC3-II protein levels, GFP-LC3 puncta and a decrease in SQSTM1 protein levels. These studies suggest that mitochondria dysfunction induced by 3-NP triggered autophagy activation. Biochemical analysis showed that 3-NP and CCCP significantly increased DRAM1 protein levels and this increase in DRAM1 played a role in 3-NP-induced autophagy activation. Although upregulation of DRAM1 by 3-NP largely depended on TP53, our present results suggested there were also other mechanisms involved [28]. The human DRAM1 gene encodes a 238 amino acid protein which acts as a stress-induced regulator of autophagy and damage-induced programmed cell death [8]. The present study demonstrated that knock-down of DRAM1 effectively blocked the 3-NP-induced induction of LC3-II and decline in SQSTM1. These studies confirm that DRAM1 plays an important role in autophagy activation.

To investigate the underlying mechanism by which DRAM1 regulates autophagy, we investigated the effects of DRAM1 on autophagosome clearance. Colocalization of EGFP-DRAM1 and LysoTracker fluorescence or DRAM1 and LAMP2 immunoflurescence confirmed predominant lysosomal localization of expressed DRAM1. We first tested if DRAM1 has an effect on autophagosome turnover following induction with rapamycin. Rapamycin can stimulate the formation of autophagosome through inhibiting mTOR. Upon removal of rapamycin, autophagosomes should be cleared if autophagy pathway is normal. The present study demonstrated that rapamycin increased the abundance of autophagosomes and the number of autophagosomes returned towards the basal levels 6 h after withdrawal of rapamycin. Knock-down of DRAM1 reduced the rate of clearance of autophagosomes after rapamycin withdrawal. Galavotti et al reported that knock-down of DRAM1 inhibited targeting of SQSTM1 to autophagosomes and reduced its degradation [34]. Our data also support the involvement of DRAM1 in degradation of autophagososmes. However, Galavotti et al found that DRAM1 was not involved in starvation- and mTOR-mediated autophagy activation [34]. Therefore, the role of DRAM1 in autophagy activation induced by other stimuli need to be further studied.

The abundance of autophagosomes is balanced by the formation and clearance of autophagosomes. After the formation, the turn-over of autophagosomes is largely determined by the process of fusion between autophagososmes and lysosomes and degradation of autophagy contents by lysosomal enzymes. mRFP-GFP tandem fluorescent-tagged LC3 showed both GFP and mRFP signal of LC3 before the fusion with lysosomes, and exhibited only the mRFP signal when LC3 transmit into lysosomes because of lysosomal acidic environment and degradation [29]. After rapamycin treatment, there was more number of mRFP-GFP-LC3 patches in non-silencing RNA-treated cells than that in DRAM1 siRAN-treated cells, suggesting DRAM1 plays a role in the formation of autophagosomes. In response to withdrawal of rapamycin, mRFP-GFP-LC3 patches quickly declined in control cells. Knock-down of DRAM1 markedly retained these mRFP-GFP-LC3 patches in the cells. These results suggest that DRAM1 stimulates clearance of autophagosomes.

Lysosomes are rich in hydrolytic enzymes and are responsible for the degradation of intracellular materials captured by autophagy [35]. After 3-NP treatment, an increase in the abundance of autophagosomes was accompanied by an increase in the number of lysosomes. The increase in acidic lysosomes was noticeable as indicated by a fluorescence dye. Knock-down of DRAM1 resulted in an impairment of lysosomal acidification and accumulation of LC3-II, indicating reduced autophagy flux. It is now generally accepted that intralysosomal low pH is maintained by an active proton pump, vacuolar H^+^­ATPases or V­ATPases. Proton transport into intracellular organelles is primarily mediated by ATP­dependent proton pumps. These pumps are therefore central to pH homeostasis in organelles. Autophagosomes and their contents are cleared upon fusing with late endosomes or lysosomes containing cathepsins, other acid hydrolases, and vacuolar [H^+^] ATPase(v-ATPase) [36], a proton pump that acidifies the newly created autolysosome. It is suggested that the proton pumps and acidification of the lysosomes were essential for the activation of lysosomal hydrolases and completion of the process of autophagy. V-ATPase may also play a role in amino acid sensing, thus plays a role in mTOR-mediated autophagy activation [37]. Inhibition of mitochondrial respiratory complex may decrease ATP production and thus decrease the activity of V-ATPase. However, due to a significant induction of DRAM1 and activation of autophagy in the present study, the V-ATPase activity was preserved to sufficiently acidify lysosomes. We speculate that DRAM1 may improve the efficiency of ATP utilization by V-ATPase. The present study found that the lower capacity for acidification of lysosomes in DRAM1 siRNA-treated cells was due to decreased V-ATPase activity. These results provide experimental data, for the first time, supporting an important role of DRAM1 in lysosomal function.

Lysosomes play important roles in autophagy. To test if the effects of DRAM1 on lysosomal functions are responsible for DRAM1-mediated autophagy activation after 3-NP treatment, the present study assessed the effects of lysosomal inhibitors on autophagosome accumulation in the presence of 3-NP. The results showed that elevating lysosomal pH and inhibiting lysosomal enzymes both increased accumulation of autophagosomes and inhibited cathepsin D activation. These results largely replicated the effects of knock-down of DRAM1 and suggested that DRAM1 probably regulated autophagy flux through lysosomes.

It should be pointed out that DRAM1 appears regulate autophagy in both early and later stages of autophagy. DRAM1 can increase the formation of autophagosomes and the clearance of autophagosomes. These effects may work through the same mechanism as DRAM1 is a lysosomal protein and may regulates dynamics of lysosomal membranes to increase V-ATPase activity and to facilitate membrane recycle for autophagosomal formation.

In conclusion, current data indicate that DRAM1 regulates autophagosome clearance through promoting lysosomal acidification and activation of lysosomal enzymes. The fusion of autophagosomes with lysosomes is an important step for autophagic degradation. In order to fully understand the role of DRAM1 in autophagy flux, the effects of DRAM1 on the fusion process between autophagosomes and lysosomes needs to be studied in the future.

## Supporting Information

Figure S1 **DRAM1 mediated autophagy activation and lysosomal acidification in Hela cells.** (A) Hela cells were transfected with DRAM1 siRNA or a non-silencing siRNA. Left: Forty-eight h after transfection of cells with DRAM1 siRNA, cells were harvested and protein levels of DRAM1 and LC3 were analyzed with immunoblotting. Right: Twenty-four hours after transfection of cells with DRAM1 siRNA, cells were treated with 3-NP (500 µM). Cells were harvested and protein levels of DRAM1 and LC3 were analyzed with immunoblotting 24 h after 3-NP. Densities of protein bands were analyzed with Sigma Scan Pro 5 and normalized to the loading control (β-actin). The data are expressed as percentage of control (non-silencing siRNA group). Bars represent mean±SE; n = 4. Statistical comparisons were carried out by ANOVA followed by Dunnett t-test. **P<0.01 (DRAM1 siRNA group vs. non-silencing siRNA group). ^##^P<0.01 (3-NP treated group vs. control group). ^$$^P<0.01 (DRAM1 siRNA group vs. non-silencing siRNA group with 3-NP treatment). (B) Representative images of GFP-LC3 fluorescence in Hela cells transfected with GFP-LC3 and treated with DRAM1 siRNAs in the presence or absence of 3-NP (500 µM). Number of cells with GFP-LC3 dots was scored in 100 GFP-LC3-positive cells. N: the nucleus. Thin arrows: GFP-LC3 dots. The scale bar represents 10 µm Bars represent mean±SE; n = 4. Statistical comparisons were carried out by ANOVA followed by Dunnett t-test. **P<0.01 (siRNA group vs. non-silencing siRNA group). (C) Lysosomal pH was measured ratio-metrically using fluorescent dextrans in Hela cells. WT Hela cells and DRAM1 siRNA1-treated cells were loaded with the pH-sensitive fluorescent dextrans by endocytosis for 1 h at 37°C and then subjected to pulse-chase assay in the presence or absence of the 3-NP (500 µM). Lanes 2 and 4 depict pH values obtained with FITC-dextran after the addition of 500 nM 3-NP. The data are expressed as percentage of control (non-silencing siRNA cells). Bars represent mean±SE; n = 4. Statistical comparisons were carried out by ANOVA followed by Dunnett t-test. **P<0.01 (DRAM1 siRNA group vs. non-silencing siRNA group). ^##^P<0.01 (DRAM1 siRNA group vs. non-silencing siRNA group with 3-NP treatment). (D) Lysosomal V-ATPase activity was inhibited in DRAM1 siRNA1-treated Hela cells. Lysosomes from control cells and DRAM1 siRNA1-treated cells were loaded with FITC-dextran (molecular weight 70,000). Hela cells were then homogenized and used for in vitro-acidification assays. Fluorescence was recorded continuously with excitation at 490 nm and emission at 520 nm. Upon addition of ATP, a progressive decrease in fluorescence intensity was observed, indicative of intralysosomal acidification. The decrement was reversed by bafilomycin A1, a V-ATPase inhibitor.
(TIF)

Figure S2 **Activity of DRAM1 antibody was blocked by DRAM1 peptide.** (A) Cells were harvested and immunoblot analysis of DRAM1 protein levels in A549 and Hela cells. Left: No peptide incubated with DRAM1 antibody before primary antibody incubation. Right: DRAM1 peptide was incubated with DRAM1 antibody for 30 min at 37°C before primary antibody incubation. (B) Cells were processed for immunofluorescence using DRAM1 antibodies (green) and DAPI (the nucleus, blue) in A549 and Hela cells, and was assessed with a confocal microscopy. Left: No peptide incubated with DRAM1 antibody before primary antibody incubation. Right: DRAM1 peptide was incubated with DRAM1 antibody for 30 min at 37°C before primary antibody incubation. N: the nucleus. Thin arrows: anti-DRAM1 fluorescence.
(TIF)
