# Supplementary figures and images for: DRAM1 Regulates Autophagy Flux through Lysosomes
Source: PLoS One. 2013 May 17;8(5):e63245. doi: 10.1371/journal.pone.0063245 (PMC3656954; doi:10.1371/journal.pone.0063245)

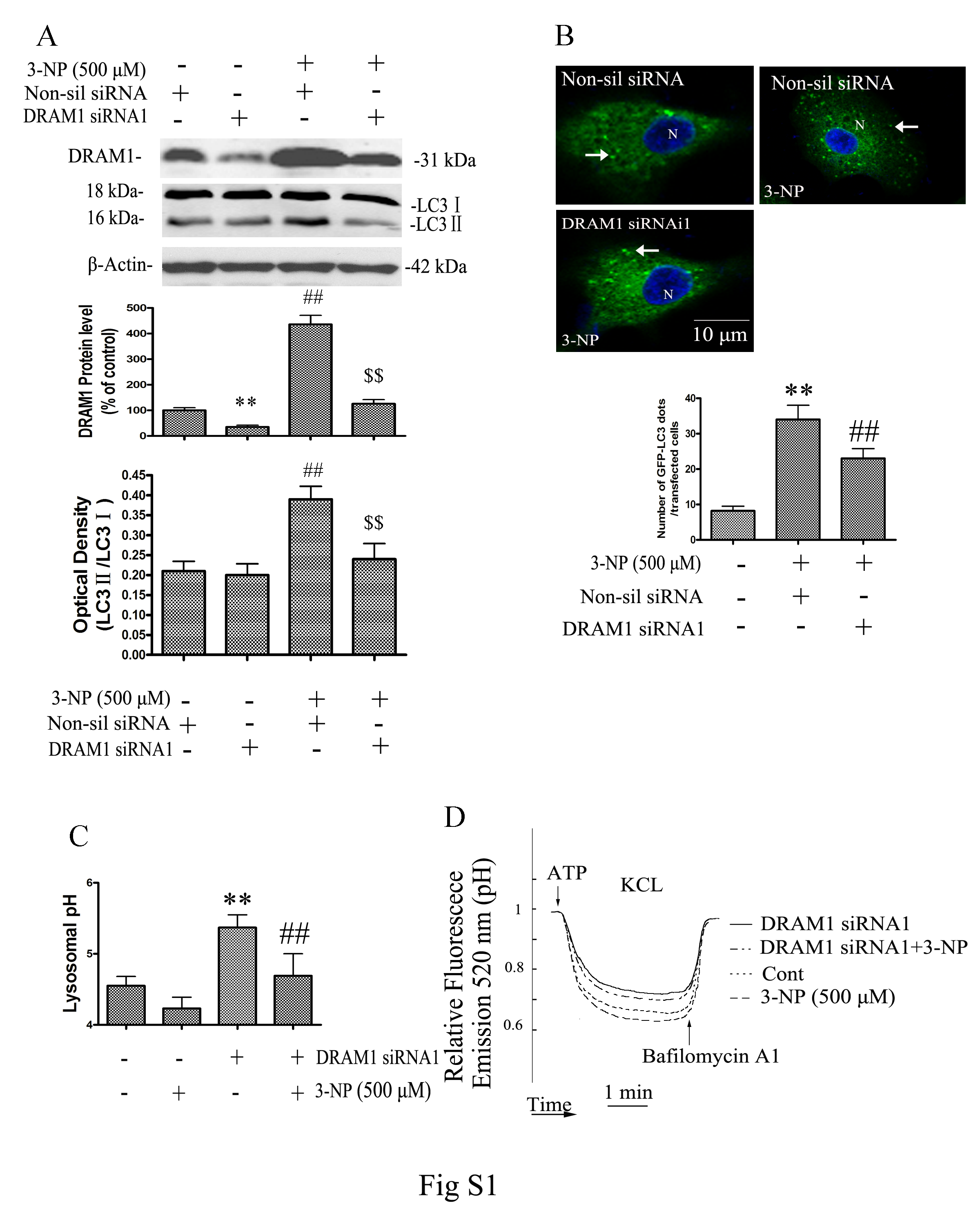

Supplement: Figure S1 — DRAM1 mediated autophagy activation and lysosomal acidification in Hela cells. (A) Hela cells were transfected with DRAM1 siRNA or a non-silencing siRNA. Left: Forty-eight h after transfection of cells with DRAM1 siRNA, cells were harvested and protein levels of DRAM1 and LC3 were analyzed with immunoblotting. Right: Twenty-four hours after transfection of cells with DRAM1 siRNA, cells were treated with 3-NP (500 µM). Cells were harvested and protein levels of DRAM1 and LC3 were analyzed with immunoblotting 24 h after 3-NP. Densities of protein bands were analyzed with Sigma Scan Pro 5 and normalized to the loading control (β-actin). The data are expressed as percentage of control (non-silencing siRNA group). Bars represent mean±SE; n = 4. Statistical comparisons were carried out by ANOVA followed by Dunnett t-test. **P<0.01 (DRAM1 siRNA group vs. non-silencing siRNA group). ##P<0.01 (3-NP treated group vs. control group). $$P<0.01 (DRAM1 siRNA group vs. non-silencing siRNA group with 3-NP treatment). (B) Representative images of GFP-LC3 fluorescence in Hela cells transfected with GFP-LC3 and treated with DRAM1 siRNAs in the presence or absence of 3-NP (500 µM). Number of cells with GFP-LC3 dots was scored in 100 GFP-LC3-positive cells. N: the nucleus. Thin arrows: GFP-LC3 dots. The scale bar represents 10 µm Bars represent mean±SE; n = 4. Statistical comparisons were carried out by ANOVA followed by Dunnett t-test. **P<0.01 (siRNA group vs. non-silencing siRNA group). (C) Lysosomal pH was measured ratio-metrically using fluorescent dextrans in Hela cells. WT Hela cells and DRAM1 siRNA1-treated cells were loaded with the pH-sensitive fluorescent dextrans by endocytosis for 1 h at 37°C and then subjected to pulse-chase assay in the presence or absence of the 3-NP (500 µM). Lanes 2 and 4 depict pH values obtained with FITC-dextran after the addition of 500 nM 3-NP. The data are expressed as percentage of control (non-silencing siRNA cells). Bars represent mean±SE; n = [file pone.0063245.s001.tif]

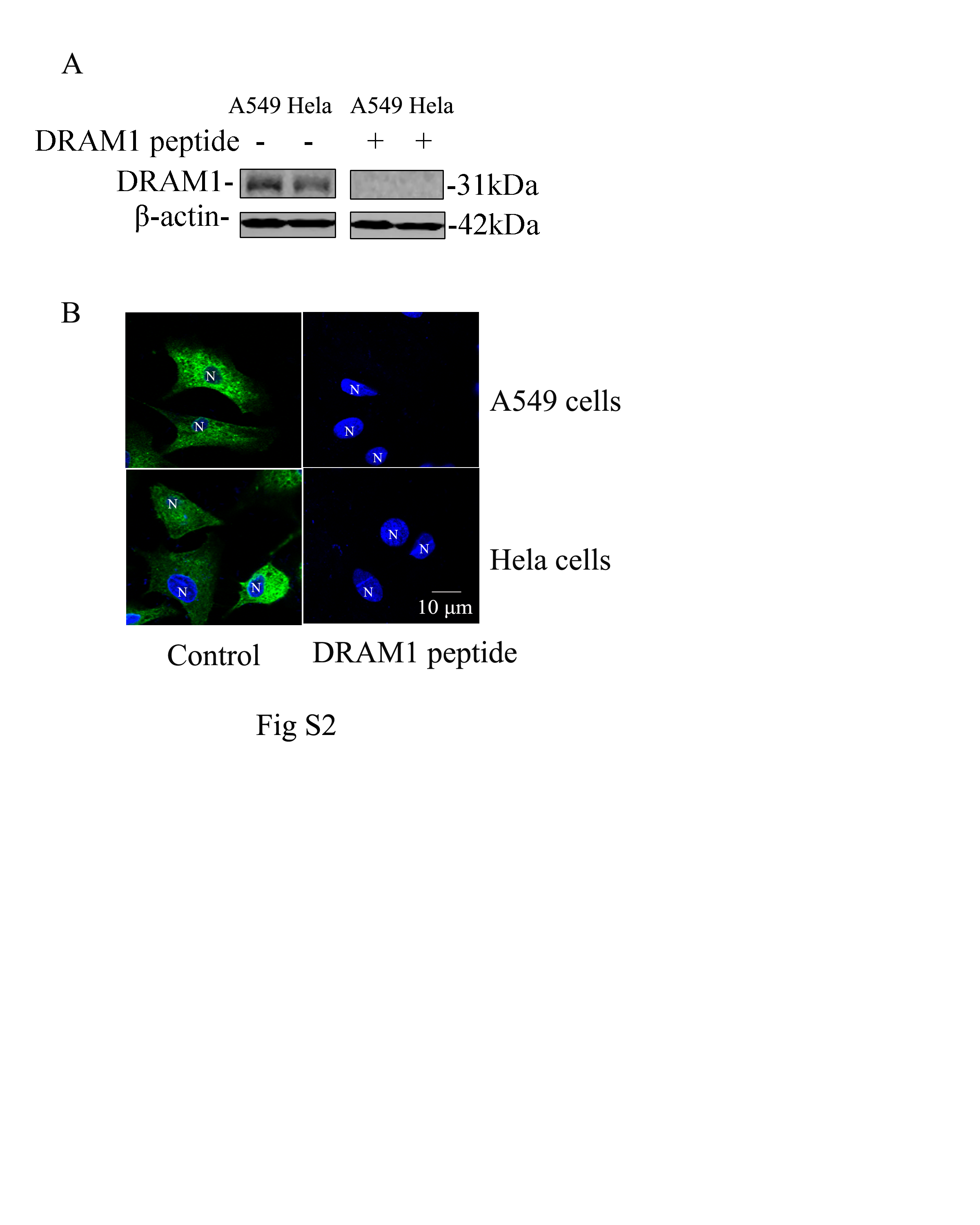

Supplement: Figure S2 — Activity of DRAM1 antibody was blocked by DRAM1 peptide. (A) Cells were harvested and immunoblot analysis of DRAM1 protein levels in A549 and Hela cells. Left: No peptide incubated with DRAM1 antibody before primary antibody incubation. Right: DRAM1 peptide was incubated with DRAM1 antibody for 30 min at 37°C before primary antibody incubation. (B) Cells were processed for immunofluorescence using DRAM1 antibodies (green) and DAPI (the nucleus, blue) in A549 and Hela cells, and was assessed with a confocal microscopy. Left: No peptide incubated with DRAM1 antibody before primary antibody incubation. Right: DRAM1 peptide was incubated with DRAM1 antibody for 30 min at 37°C before primary antibody incubation. N: the nucleus. Thin arrows: anti-DRAM1 fluorescence. (TIF) [file pone.0063245.s002.tif]
